# Inter‐Stimulus‐Interval Dependence in Auditory Cortex Reflects Perceptual Organization of Streams Under Informational Masking

**DOI:** 10.1111/psyp.70198

**Published:** 2025-11-30

**Authors:** Clara Raudonat, Eva Doroszewski, Alexander Gutschalk

**Affiliations:** ^1^ Department of Neurology Ruprecht‐Karls‐Universität Heidelberg Heidelberg Germany

**Keywords:** auditory perception, awareness related negativity, informational masking, magnetoencephalography, N1, selective attention

## Abstract

The perceptual saliency of a regular sound sequence embedded into a well‐chosen multi‐tone‐masker background fluctuates despite the absence of change in the physical stimulus. When such tone streams are perceived, each tone of the stream evokes a negative‐going response referred to as awareness‐related negativity (ARN). Here we probe if the ARN amplitude shows a dependence on the inter‐stimulus interval (ISI) within perceived tone streams, similar to the N1 response evoked by unmasked tone streams independently of a task. Isochronous target streams were presented under multi‐tone masking with ISIs of 500, 850, and 1200 ms, while listeners indicated when they perceived the onset of the target streams. A control experiment applied the same tone streams without masking in the presence of a second, narrow‐band noise stream. In two separate runs, listeners either attended the tone streams and indicated their beginning and ending, or they attended the distractor streams, in which they performed a deviance detection task. Activity in auditory cortex was recorded with magnetoencephalography. Experiment 1 revealed a significant amplitude increment of the ARN with increasing ISI. In Experiment 2, the known amplitude dependence of the N1 was observed for attended and unattended runs alike. Critically, the negative difference waveform (Nd) between attended and unattended runs did not increase with the ISI. These results support an interpretation of the ARN as being related to the neural formation of an auditory stream and not as a pure reflection of attentional response enhancement.

## Impact Statement

Mechanisms of auditory scene analysis and attention mechanisms have been independently attributed as mechanisms for perceptual awareness of tone streams. Here we show that the dependence of auditory cortex activity on the inter‐stimulus interval is independent of attention when the stimuli are not masked. Conversely, when presented under masking, inter‐stimulus‐interval dependence was only observed when listeners were aware of the streams' presence, dissociating this awareness‐related activity from the negative difference wave reflecting attentional enhancement.

## 1 Introduction

Serial auditory events are perceptually organized into coherent auditory streams, depending on their similarity, repetition rate, and context in the auditory scene (Bregman 1990). For example, sequences of repeated tones can form auditory streams that prominently stand out when presented with a background of random tones. In other instances, the sequence disappears within the random tones and remains undetectable, which is known as informational masking (Kidd Jr. et al. 1994, 2003). Note that informational masking is not only used to describe the random multi‐tone maskers described here, but also for multiple other contexts, for example, masking of speech by speech (Brungart et al. 2001), with the common feature that the masking cannot already be explained by energetic masking in the cochlea (Durlach et al. 2003). Whether a sequence is perceived or masked depends on the sequence and masker structure (Kidd et al. 2008), but there is an ambiguous range where the target stream can be variably perceived or masked in different trials (Gutschalk et al. 2008; Micheyl et al. 2007). Such ambiguous stimuli have been applied in magnetoencephalography (MEG), and it was shown that the evoked response elicited by each single tone of the target stream was prominently stronger once the stream was perceived compared to when it was masked (Gutschalk et al. 2008). The negative‐going response in auditory cortex evoked by the perceived tones of a target stream has therefore been labeled as awareness‐related negativity (ARN).

For the simpler stream segregation paradigm, where the grouping of two serially presented tones depends on parameters such as rate and frequency difference, a relationship between frequency‐selective adaptation and stream segregation has been discussed based on recordings in primate auditory cortex (Fishman et al. 2001; Micheyl et al. 2005; Banno et al. 2023). In non‐invasive EEG and MEG recordings from human auditory cortex, it was found that tone‐evoked P1, N1, and P2 responses are stronger for stimuli that are more likely perceived as two streams because of a higher frequency difference, and that the response strength is related to the inter‐stimulus interval (ISI) within a tone stream (Gutschalk et al. 2005; Snyder et al. 2006, 2009; Gutschalk, Oxenham, et al. 2007; Alain et al. 2017).

A monotonic ISI dependence of the N1 response is well known from single‐stream setups in a range of 300 ms up to multiple seconds (Ritter et al. 1968; Hari et al. 1982; Imada et al. 1997). The N1 has also been known to adapt selectively to the previous tone (Butler 1968; Näätänen et al. 1988), depending on the frequency difference between subsequent tones. However, it is unlikely that the N1 reflects the same early processing levels studied in primate models, the latter of which were mostly made from early‐latency neurons in middle cortical layers (Fishman et al. 2001). The N1 and ARN are more likely generated by synapses in superficial cortical layers, reflecting input into apical dendrites of layer 5 neurons (Kohl et al. 2022; Fernandez Pujol et al. 2023).

The level of processing of the N1 might thus be more closely related to that of the mismatch negativity (MMN), which has been previously shown to operate on perceived streams (Sussman et al. 1999; Winkler et al. 2009). Under multi‐tone masking, the MMN to tone deviants is only observed when the standard tones are perceived as a distinct stream and evoke an ARN themselves (Dykstra and Gutschalk 2015). We therefore propose to transfer the concept of ISI‐dependent adaptation within streams (Gutschalk et al. 2005) to the perception of streams under multi‐tone masking, and propose that a similar ISI effect is observed for the ARN as has long been known for the N1 in the absence of sensory competition (Hari et al. 1982).

For most cases, the detection of auditory streams under multi‐tone masking is supported by attention. It has been demonstrated that attention enhances both, the likelihood of the stream's perception, as well as the associated negative‐going responses in auditory cortex (Gutschalk et al. 2008; Elhilali et al. 2009; O'Sullivan et al. 2015; Molloy et al. 2019; Doll et al. 2024). However, selective attention can also be deployed to readily segregated streams, without changing the perceptual organization: When the N1 is obtained under selective attention, the overall negative‐going response is enhanced, partly overlapping the N1 (Hillyard et al. 1973) and extending to longer‐latency ranges (Näätänen et al. 1978). This enhancement for attended over unattended stimuli has received different labels, the most pragmatic being the negative difference wave (Nd), which we will adopt here (Hansen and Hillyard 1980). The Nd increases with the frequency difference between target and distractor (Alain et al. 1993), and is not observed when the two are too close (Alain and Woods 1994), supposedly because the Nd is also constrained by the formation of auditory streams.

The influence of the ISI on the Nd is more complex than for the N1: it has been proposed (Schwent et al. 1976) that a high stimulus rate (ISI < 500 ms) was necessary to generate an early Nd that overlaps with the N1, whereas at longer ISIs, around 800 ms and more, the Nd occurred subsequent to the N1 (Näätänen et al. 1978). Thus, in the early time range up to around 100–150 ms, the Nd did not increase with the ISI (Näätänen et al. 1981; Hansen and Hillyard 1984) but in a latency range beyond 200 ms, the Nd increases with the ISI. However, when the ISI was compared between 480 and 800 ms, there was no significant increase in the late Nd, while the same ISI range had a prominent effect on the amplitude of the N1 response, when the same stimuli were not task relevant (Hansen and Hillyard 1984). In summary, while the N1 appears to monotonically increase with the ISI, the early part of the Nd that overlaps with the N1 does not increase with the ISI and may even be absent at longer ISI.

Here, based on the hypothesis that the N1 reflects the temporal organization of auditory streams, we expected that a similar ISI dependence should be observed for the ARN evoked by isochronous tone streams once they are detected in the presence of a multi‐tone informational masker. No target‐stream ISI dependence of the response evoked by the same tones was expected when they are not grouped into a coherent stream and supposedly group together with the masker tones, instead. In this case, we expected a response resembling the one evoked by masker tones (Dykstra and Gutschalk 2015), which is determined by masker parameters rather than the ISI within the target stream. To test the hypothesis that the ARN shows N1‐like ISI dependence, we recorded the ARN with three different target‐stream ISIs, all embedded in the same random multi‐tone masker. As reference for the N1 and Nd responses, a similar target stream was presented in combination with a second stream that did not mask the target stream. These two streams were configured to ensure that perceptual organization was unambiguous and remained stable, regardless of which stream was attended. Accordingly, we expected that the N1 increased with the ISI for both, attended and unattended trials, whereas the early Nd was not expected to covary with the ISI. Such a dissociation of the Nd and ARN would support models that do not generally require attention for auditory perceptual awareness (Sussman et al. 2007; Dykstra et al. 2017; Dembski et al. 2021), but rather consider attention a modulating factor for perception. In contrast, if perceptual awareness of tone streams in the presence of a multi‐tone masker was exclusively explained by attention, we would expect highly similar behavior of the ARN and Nd, and thus would not expect any ISI dependence of the ARN that was not similarly observed for the Nd in the control experiment.

## 2 Methods

### 2.1 Listeners

Eighteen adults (6 male and 12 female) participated in both experiments. Age ranged from 23 to 35 years (mean age 26 years). Only one of the listeners was left‐handed. All participants had normal hearing according to self‐report. Some of the listeners had already taken part in other MEG experiments. All participants provided written informed consent before participating in this study, which was approved by the medical faculty's review board. No data set was excluded from the analysis. The ISI dependence of the ARN was expected to be at least of similar size as the lateralization effect reported in a previous study in the left auditory cortex (Königs and Gutschalk 2012), where the standard deviation amounted to about 1.1 times the amplitude difference, using otherwise similar methods as in the present study. Based on these data, a sample size of 12 would provide a power of 0.82 and a sample size of 18 would provide a power of 0.95 (alpha two‐sided 0.05; *t*‐test for repeated measures).

### 2.2 Stimuli and Task

**FIGURE 1:**
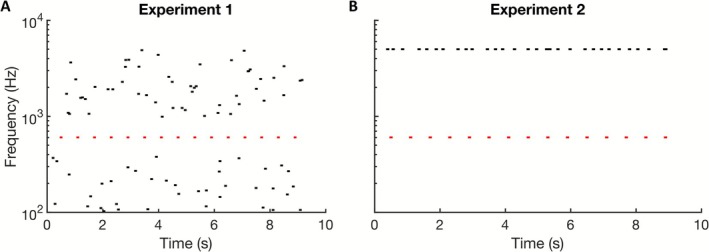
(A) Schematic spectrogram of the informational‐masking paradigm used in experiment 1, where an isochronous target (red) is presented in the middle of a random multi‐tone masker (black) ranging from 100 to 5000 Hz. In the example, the target stream consists of 1133‐Hz tones repeated with an ISI of 500 ms. A protected region around the target stream amounted to ± half an octave. (B) In experiment 2, the same target stream (red) was presented together with a distractor stream of repetitive narrow‐band noise bursts with a center frequency of 5000 Hz (black). This stream comprised up to two amplitude modulated events, which were used as targets in the distraction task.

The same target sequences were either embedded in a multi‐tone masker (Figure 1A) or presented together with a second stream of narrow‐band noise bursts (Figure 1B). Each target‐tone sequence was chosen from five different frequencies on a log scale (603, 826, 1133, 1553, and 2129 Hz) and three different ISIs (500, 850, and 1200 ms). The trial duration was fixed, and the number of tones per sequence differed between ISIs, amounting to 15 for the 500‐ms ISI, 10 for the 850‐ms ISI, and 7 for the 1200‐ms ISI. The duration of all masker and target tones amounted to 100 ms, including 5‐ms long raised cosine ramps at the beginning and ending (i.e., the stimulus onset asynchrony (SOA) of the three ISI conditions amounted to 600 ms, 950 ms, and 1300 ms). All stimuli were created with a 48 kHz sample rate and 32‐bit digitization depths in MATLAB.

In experiment 1, participants were instructed to press a response button as soon as they detected a regular target stream. Moreover, they were instructed to follow the target stream and, in case they lost it or had pressed the button in error, were instructed to press the button once again. The target was presented along with a random multi‐tone masker with a frequency range from 100 to 5000 Hz, randomized on a logarithmic scale. A protected region of half an octave on each side of the target frequencies was chosen to reduce energetic masking. The multi‐tone masker was generated in 300‐ms‐long time windows with 5 tones per time segment. The timing of the masker tones was jittered ±150 ms. To match the duration between target and masker, a slightly different total number of masker segments was used (30, 32, and 31 segments for ISIs of 500, 850, and 1200 ms, respectively), amounting to total durations of 9, 9.6, and 9.3 s. Stimuli with a specific frequency and ISI were presented to the participants in a randomized order. 25 catch trials without any target stream were included in the paradigm. The target streams were started with the center of the first masker interval, that is, up to 150 ms after the onset of the first masker tone. To equalize the number of averaged tones per ISI condition, the 500‐ms ISI was presented 25 times (5 times for each frequency), the 850‐ms ISI 35 times (7 times for each frequency), and the 1200‐ms ISI was repeated 55 times (11 times for each frequency).

In experiment 2, the same pure‐tone target sequences as in experiment 1 were presented together with a distractor sequence, which consisted of narrow‐band noise bursts at a constant center frequency of 5000 Hz and started and ended approximately together with the target tone sequences. This distractor sequence was made dissimilar to the target sequences, such that it caused virtually no informational masking. The noise bursts were generated by filtering broadband noise with 4th‐order low‐ and high‐pass Butterworth filters with a cutoff frequency of 5000 Hz; they were 100 ms long, ramped on and off with 5‐ms‐long cosine ramps, and were presented with a random ISI between 50 and 350 ms. In each sequence, 0, 1, or 2 of the noise bursts were amplitude‐modulated (AM) deviants with a frequency of 60 Hz and a modulation depth of 70%. These deviants served as targets when listeners were instructed to attend to the distractor sequence. In trials with two deviants, one AM deviant occurred randomly within the first half of the noise sequence and another deviant within the second half.

To equalize the number of tones per ISI condition in the target sequences, the pure‐tone sequences with 500‐ms ISI were repeated 30 times (2 repetitions at 5 different frequencies), the 850‐ms ISI 45 times (3 repetitions at 5 different frequencies), and the 1200‐ms ISI 75 times (5 repetitions at 5 different frequencies). Furthermore, 18 shorter trials were included as deviants (2 repetitions for all three ISIs for frequencies 603, 1133, and 2129 Hz), which ended before the noise sequence. These early‐ending deviants served as targets when listeners were instructed to attend to the tone sequences (i.e., to the target sequences). These shorter tone sequences comprised 13 tones for the 500‐ms ISI, 8 tones for the 850‐ms ISI, and 6 tones for the 1200‐ms ISI.

Experiment 2 was recorded in a second session and with two different runs, where participants were either instructed to attend to the tone sequences or to the noise sequences: When instructed to attend to the tone sequences, participants were asked to press a response button as soon as a tone sequence started. Moreover, they were instructed to press the response button, once again, when the tone sequence ended before the noise sequence, that is, when they detected an early‐ending within the tone sequence. When instructed to attend to the noise sequences, the participants were asked to press the response button each time an AM deviant occurred within the stream of narrow‐band noise bursts.

Both experiments were presented in pseudorandomized order; 8 of the participants started with experiment 2 of which half started with attending tone sequences and half started with attending narrow‐band noise sequences. The remaining 10 participants started with experiment 1 followed by experiment 2, which again was started by one half with attending tone sequences and the other half with attending narrow‐band noise sequences. Furthermore, 11 participants completed all three sub‐experiments in two sessions (A and B) on the same day, while the rest split the sessions over two different days.

### 2.3 MEG Acquisition

The data were recorded in a magnetically shielded room with a Neuromag‐122 whole‐head MEG system. Stimuli were presented via ER3 earphones connected by foam earplugs. Presentation was binaural with a level of approximately 65 dB SPL for the single pure tone (measured for continuous pure tones with a 2 cc coupler). This holds for target and masker tones, although masker‐tone intensity was increasingly attenuated from about 2000–5000 Hz by 20 dB. The narrow‐band noise around 5000 Hz used in experiment 2 was presented with a lower sound intensity of approximately 47 dB, but was still well audible. For two participants, the overall level was increased by 3 dB for convenience. Before MEG measurements, training was performed with all participants, once with pure‐target tones and once with the target stream presented within the masker.

The measurements were recorded directl coupled using a 1000 Hz sampling rate and a low pass recording filter at 330 Hz. Before MEG recording, four position‐indicator coils were fixed to the participants' heads. Afterwards, the head was digitized with reference to the nasion, and the two preauricular points along with 100 points on the head surface. The head position relative to the dewar was then determined by measuring the position of the indicator coils before each recording.

### 2.4 Data Analysis

In the analysis of experiment 1, all responses made between two tones of the target stream and up to 1000 ms after the last tone were considered. All responses made during catch trials were considered false alarms. The tone‐wise detection rate included up to two tones before the button press as detected, considering that detection of the target stream required at least one tone repetition.

In experiment 2, responses indicating the tone stream were considered up to 1000 ms after the first tone. Responses indicating an early ending were considered up to 1000 ms after the last tone in the sequence. When the noise bursts were attended, correct detections of AM target bursts were considered up to 1000 ms after tone onset. All other responses were considered false alarms.

The analysis was carried out using Brain Electric Source Analysis (BESA Version 6.1, BESA software GmbH, Germany). Evoked fields were averaged over target tones in both experiments. Artifact epochs were identified by an automatic amplitude and threshold criterion. In experiment 1, tones were separately averaged for detected and undetected intervals, including the two tones prior to a button press as detected. An epoch from −500 to 1200 ms and a baseline from −100 to 0 ms were assigned for all conditions. Spatiotemporal dipole source analysis was performed in the following way: Three PCA components were defined on the raw data for modeling low‐frequency artifacts (mostly caused by distant street cars). Moreover, two regional sources were placed over the eyes to model blink and eye‐movement artifacts. Finally, two dipoles (one per auditory cortex) were simultaneously fitted to the first negative peak of a grand average across all three ISI conditions, separately in both experiments. This procedure was repeated for each individual participant. For experiment 1, the average of detected trials was used, while for experiment 2 the attended tone streams were used for dipole fitting.

The dipole and artifact models were then applied to all conditions to derive source waveforms in the auditory cortex. These data were then transferred to MATLAB for further analysis, where the data were filtered with a 2nd‐order low‐pass filter with a cutoff frequency of 20 Hz (zero‐phase‐shift Butterworth filter).

The time intervals, in which the ARN and N1 amplitudes were measured, respectively, were assigned based on the grand‐average source waveforms, separately for experiments 1 and 2. First, the peak latency was measured for each condition (3 ISIs and 2 hemispheres) in the grand average across participants, and then the mean across conditions was calculated. The measurement time interval was then defined 15 ms before and after the average peak amplitude. This finally resulted in a time interval of 105–135 ms for experiment 1 and an interval of 85–115 ms for experiment 2.

Additional, exploratory analyses were performed in the following time intervals: To evaluate specifically the Nd in Experiment 2, the time interval 15 ms before and after the average latency of the difference‐response peak was evaluated in the time interval 98–128 ms. To evaluate the behavior of a second negative‐going response component, the time intervals 180–210 ms in experiment 1, and 200–230 ms in experiment 2 were evaluated.

The amplitude was then measured as mean amplitude in this time interval in the single‐participant waveforms for the left and right auditory cortex and each condition. Time intervals rather than peaks were measured, because this procedure allows measuring the same time range also for missed trials, in which clear peaks can often not be identified. Statistical analysis was performed using SPSS (IBM SPSS Statistics 27). First, the data were tested for normal distribution using the Shapiro–Wilk test. When testing for normal distribution, one participant (number 6) in the first experiment and two participants (number 6 and 13) in the second experiment stood out as outliers. When their data were excluded, the data showed a normal distribution according to the Shapiro–Wilk test, *p* > 0.05, for both detected and missed tones in the first experiment and for attended and unattended tones in the second experiment. On closer inspection, both participants showed high amplitude, which, however, exhibited the same tendency across all conditions as the other participants.

Statistical analysis of amplitudes was performed with repeated‐measures ANOVA with three different factors: detected/missed (Experiment 1) and attended/unattended (Experiment 2), respectively, ISI (500/850/1200), and hemisphere (right and left). The effect of ISI was evaluated with a linear contrast analysis including linear and quadratic trends. The analysis was limited to these two options, because they can model the monotonic increment that was expected for response amplitude in dependence on the ISI. Our hypothesis was that this increment was linear, but quadratic trends were also evaluated to observe potential saturation of ISI dependence. Higher order contrasts were not evaluated, since they were not considered meaningful for our research question. When the evaluation was performed with and without the outliers, no meaningful difference was observed in the statistical analysis. The analysis including all participants is therefore reported in the results section.

## 3 Results

### 3.1 Experiment 1

**FIGURE 2:**
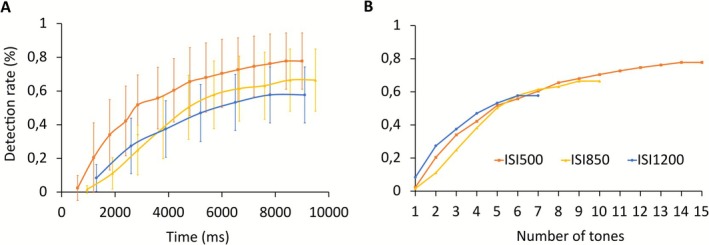
(A) Mean (±standard deviation) detection rate of isochronous target streams averaged across participants (*n* = 18). The build‐up is separately shown for the 500‐ms (orange), 850‐ms (blue), and 1200‐ms ISIs (yellow). Shorter ISI is associated with on average higher detection rate by the end of the sequence. (B) When the same data are plotted over tone number in the sequence, instead, a more similar build‐up is observed across ISI conditions.

Tone‐wise detection rates in the first experiment showed a significant dependence on the ISI (F_2,51_ = 5.316; p = 0.008; $\eta_{p}^{2}$ = 0.173): when counting two tones before the button press as detected, the rate of detected tones relative to all tones was highest for the smallest ISI (500 ms) at 57% (±16%), it decreased for the 850‐ms ISI to 44% (±15%), and further for the 1200‐ms ISI to 41% (±14%; mean ± standard deviation). Accordingly, when the tone‐wise detection rate is plotted relative to the time elapsed (Figure 2A), the built‐up is faster and the detection rate by the end of the sequence is higher for the short ISI. The latter, trial‐wise hit rates were 74% (±17%) for ISI 500 ms, 65% (±18%) for ISI 850 ms, and 55% (±15%) for ISI 1200 ms. When the comparison is based on the number of tones (Figure 2B) instead, i.e., when only tones 1–7 were considered, there was no significant difference between the three ISIs (*F*_2,51_ = 0.795; *p* = 0.457; $\eta_{p}^{2}$ = 0.031). The trial‐wise false alarm rate for the first experiment amounted to 30.3% ± 15.6% (mean ± standard deviation).

**FIGURE 3:**
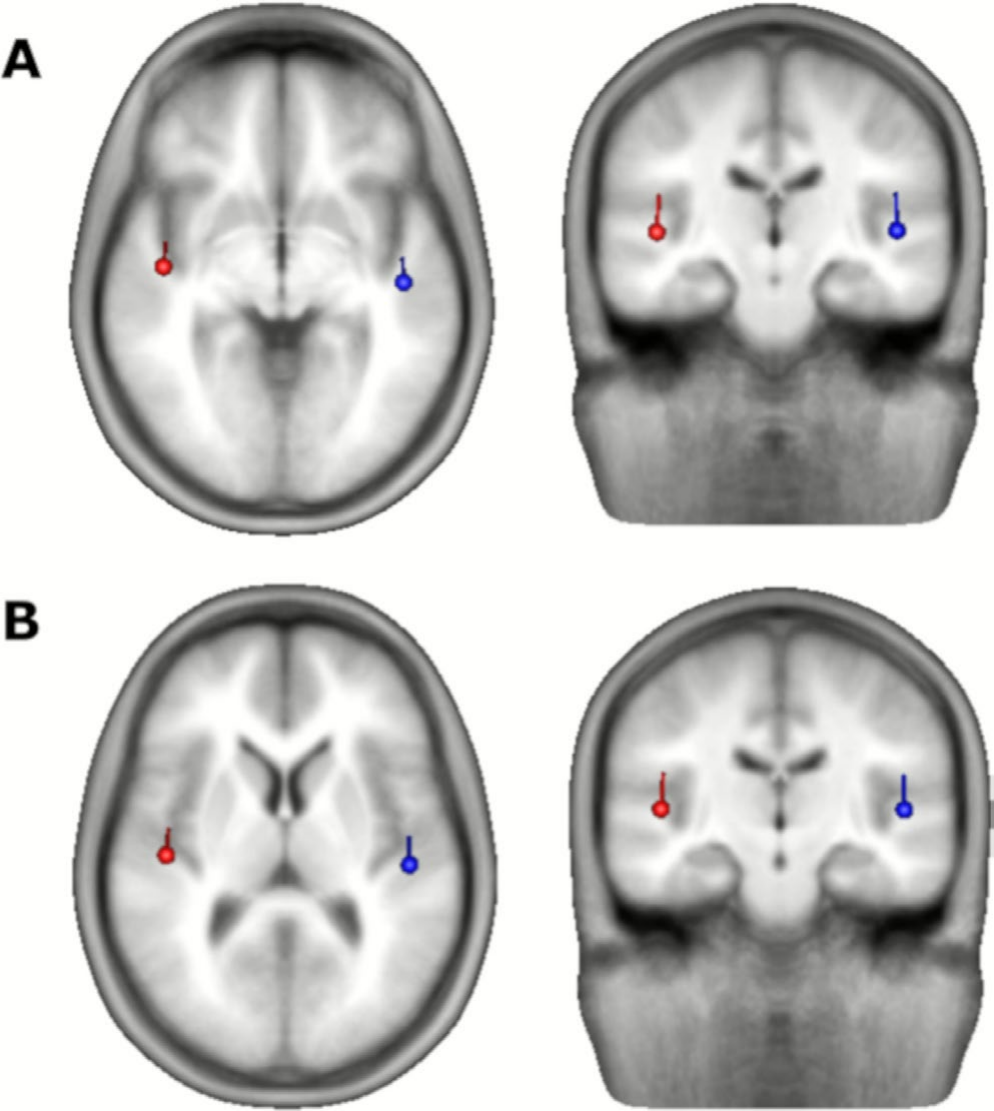
Dipole locations in auditory cortex averaged over all participants (*n* = 18) in an approximated Talairach space. Dipoles were fitted to the ARN in experiment 1 (A) and to the N1 in experiment 2 (B), in both cases averaged across all three ISI conditions.

**FIGURE 4:**
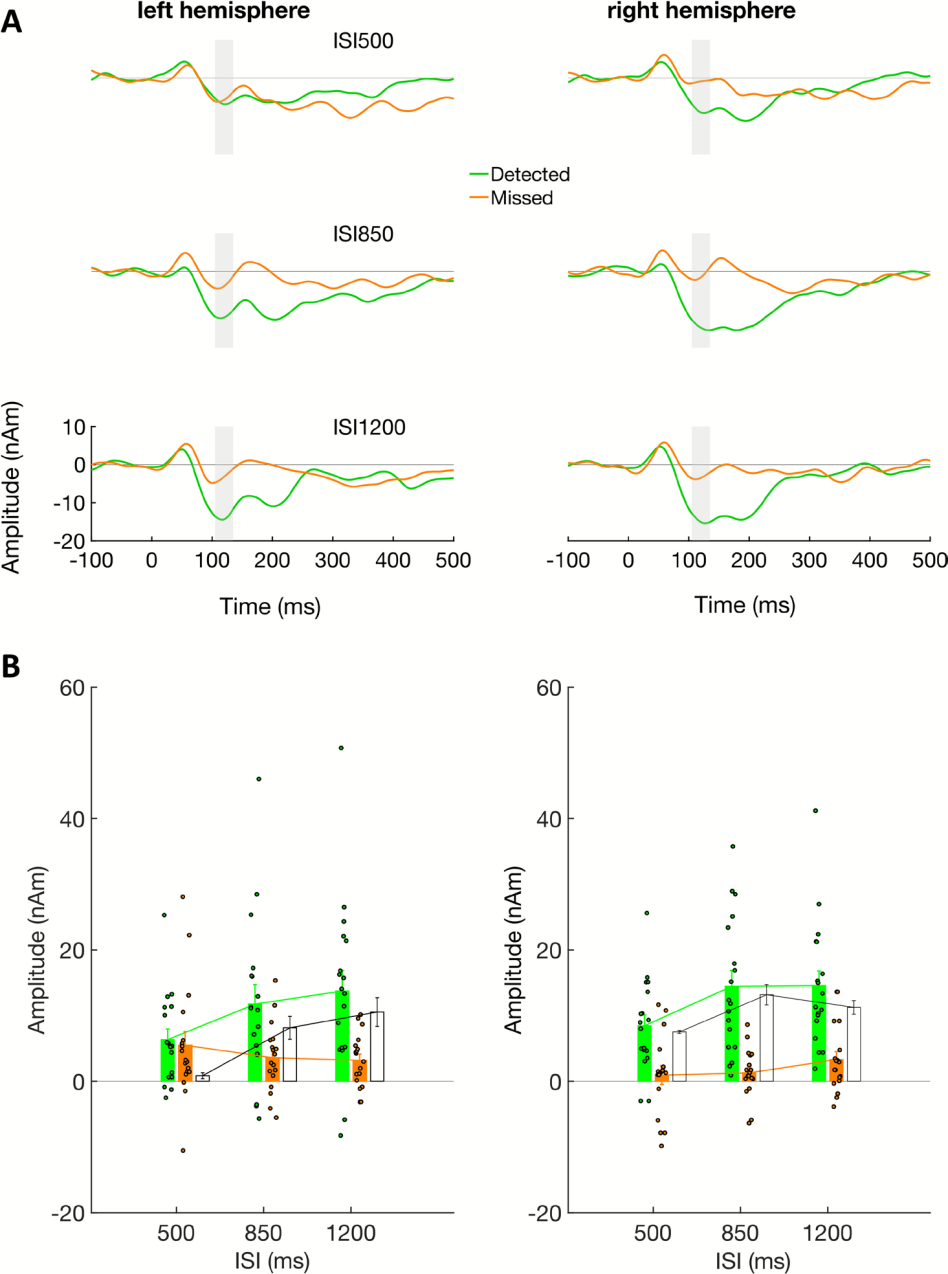
(A) Grand average (*n* = 18) source waveforms of Experiment 1 for dipoles in left and right auditory cortex. The three ISI conditions (500, 850, and 1200 ms) are plotted on the *x* axis. The source waveforms are separately plotted for detected (green) and undetected (orange) trials. The time interval in which the ARN amplitude was measured for the analysis (105–135 ms) is shaded in gray. (B) Mean (± standard error) amplitudes in the left and right auditory‐cortex source waveforms across participants (*n* = 18) in the ARN interval (105–135 ms) for experiment 1; single participant amplitudes indicated as small circle. The values are separately plotted for hit (green) and miss (orange) trials over the three ISI conditions used.

The MEG analysis was based on source waveforms derived from dipole sources in auditory cortex (Figure 3A). As in previous studies, detected tones evoked a broad‐based negative‐going response from approximately 75–275 ms (Figure 4A), which was significantly stronger than the response in undetected epochs. Statistical results are summarized in Table 1; based on the grand‐average peak latency, the amplitude of the ARN was measured in the time interval 105–135 ms. In this time range, the ARN increased with the ISI, showing a significant linear but no quadratic effect (Table 1b). The growth was more prominent from the 500‐ to the 850‐ms ISIs (*F*_1,17_ = 11.299; *p* = 0.004; $\eta_{p}^{2}$ = 0.399), and not significant from 850 to 1200 ms (*F*_1,17_ = 0.700; *p* = 0.415; $\eta_{p}^{2}$ = 0.040; post hoc tests not corrected for multiple comparison). For the undetected tones, in turn, only a small negative‐going wave was evident for all three ISIs, which showed neither a significant linear nor a quadratic effect with ISI (Table 1c). Accordingly, there was also a linear interaction of detection × ISI in the linear contrast analysis (Table 1a), confirming the main hypothesis that the ARN shows ISI dependence. An unexpected detection × hemisphere interaction was driven by stronger left‐hemisphere responses for undetected tones, whereas no significant hemisphere effect was observed for detected tones.

**TABLE 1:** Statistical analysis of Experiment 1. (a) ANOVA for repeated measures for the ARN amplitude in the source waveforms (105–135 ms) with the factors detection (detected, undetected), ISI (500, 850, 1200 ms; linear contrast analysis), and hemisphere (left, right) (b) ANOVA for detected tones, only (c) ANOVA for undetected tones, only.

	Contrast	df	*F*	Sig.	$\eta_{p}^{2}$
*(a) Detected vs undetected*					
Detection		1,17	20.500	< 0.001	0.547
ISI	Linear	1,17	9.844	0.006	0.367
	Quadratic	1,17	0.818	0.379	0.046
Hemisphere		1,17	0.024	0.880	0.001
Detected*ISI	Linear	1,17	5.832	0.027	0.255
	Quadratic	1,17	3.055	0.099	0.152
Detected*Hemisphere		1,17	7.088	0.016	0.294
Detected*ISI*Hemisphere	Linear	1,17	3.354	0.085	0.165
	Quadratic	1,17	0.697	0.416	0.039
*(b) Detected*					
ISI	Linear	1,17	18.121	< 0.001	0.516
	Quadratic	1,17	3.369	0.084	0.165
Hemisphere		1,17	0.976	0.337	0.054
ISI*Hemisphere	Linear	1,17	0.340	0.567	0.020
	Quadratic	1,17	1.161	0.296	0.064
*(c) Undetected*					
ISI	Linear	1,17	0.000	0.984	0.000
	Quadratic	1,17	0.445	0.514	0.026
Hemisphere		1,17	9.215	0.007	0.352
ISI*Hemisphere	Linear	1,17	3.888	0.065	0.186
	Quadratic	1,17	0.005	0.944	0.000

### 3.2 Experiment 2

In the control experiment 2, the detection rate for the tonal target stream was at ceiling with 97% ± 4.2% (mean ± standard deviation), which was expected since the tonal stream was not masked by the noise‐burst sequence. The early ending of control trials was detected on average in 72% ± 27% (mean ± standard deviation), with a low false alarm rate (5% ± 7%; relative to the standard trials), confirming that participants attended to the tone streams.

To distract attention from the tone streams, listeners were instructed to attend to the higher‐frequency noise bursts in an alternative run, and to detect rare, low‐saliency AM deviants within this distractor stream. The detection rate for amplitude‐modulated deviants in the distraction task was 79% ± 15% (mean ± standard deviation); the false alarm rate amounted to 1% ± 2% (scaled to the number of deviants), indicating overall a similar level of difficulty for both tasks. Note that the physical stimuli were identical in both runs, and that the order in which the tasks were performed was balanced between participants.

**FIGURE 5:**
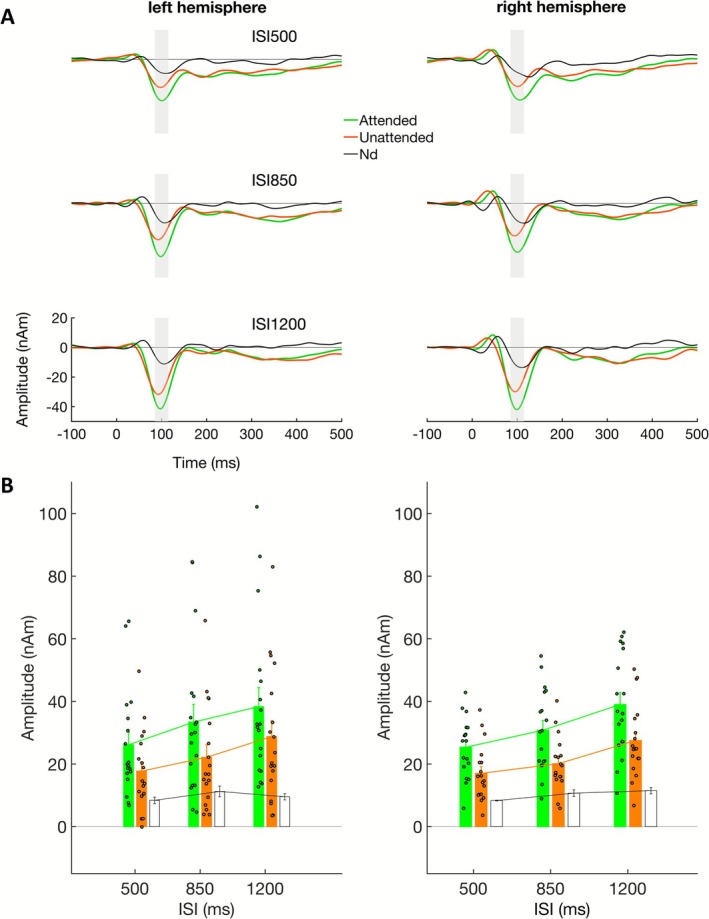
(A) Grand average (*n* = 18) source waveforms of Experiment 2 for dipoles in left and right auditory cortex. The three ISI conditions (500, 850, and 1200 ms) are plotted in a column. The source waveforms are separately plotted for attended (green) and unattended (orange) trials. The difference of attended minus unattended is plotted in black. The time interval in which the N1 amplitude was measured for the analysis (85–115 ms) is shaded in gray. (B) Mean (± standard error) N1 amplitudes in the left and right auditory‐cortex source waveforms across participants (*n* = 18) in experiment 2 (85–115 ms); single participant amplitudes indicated as small circle. The values are separately plotted for attended (green) and unattended (orange) trials over the three ISI conditions used. Mean difference amplitudes are indicated as white bars.

The dipole fit showed a source location of the N1 in auditory cortex (Figure 3B), consistent with the source of the ARN (Figure 3A). The source waveforms (Figure 5A) show that the tone stream evoked larger responses when task‐relevant and attended, compared to when the distant noise bursts were task relevant. In the time interval 85–115 ms (Figure 5B), around the grand average N1 peak latency, this attention effect was highly significant (cf. Table 2 for statistical results). Moreover, there was a highly significant effect of the ISI, but no significant linear or quadratic interaction of ISI * attention (although a significant quadratic effect was observed for unattended but not for attended). No significant main effects or interactions with hemisphere were observed. The overall ISI effect was observed in both intervals (500–850 ms: *F*_1,17_ = 11.358; *p* = 0.004; $\eta_{p}^{2}$ = 0.401; 850–1200 ms: *F*_1,17_ = 50.794; *p* < 0.001; $\eta_{p}^{2}$ = 0.749).

**TABLE 2:** Statistical analysis of Experiment 2. (a) ANOVA for repeated measures for the N1 amplitude in the source waveforms (85–115 ms) with the factors attention (attended, unattended), ISI (500, 850, 1200 ms; linear contrast analysis), and hemisphere (left, right) (b) ANOVA for attended tones, only (c) ANOVA for unattended tones, only.

	Contrast	df	*F*	Sig.	$\eta_{p}^{2}$
*(a) Attended vs. unattended*					
Attention		1,17	18.442	< 0.001	0.520
ISI	Linear	1,17	33.465	< 0.001	0.663
	Quadratic	1,17	1.482	0.240	0.080
Hemisphere		1,17	0.090	0.768	0.005
Attention*ISI	Linear	1,17	2.124	0.163	0.111
	Quadratic	1,17	1.452	0.245	0.079
Attention*Hemisphere		1,17	0.074	0.789	0.004
Attention*ISI*Hemisphere	Linear	1,17	1.095	0.310	0.060
	Quadratic	1,17	0.831	0.375	0.047
*(b) Attended*					
ISI	Linear	1,17	28.920	< 0.001	0.630
	Quadratic	1,17	0.009	0.925	0.001
Hemisphere		1,17	0.059	0.812	0.003
ISI*Hemisphere	Linear	1,17	0.441	0.515	0.025
	Quadratic	1,17	4.286	0.054	0.201
*(c) Unattended*					
ISI	Linear	1,17	31.322	< 0.001	0.648
	Quadratic	1,17	9.640	0.006	0.567
Hemisphere		1,17	0.117	0.736	0.007
ISI*Hemisphere	Linear	1,17	0.088	0.771	0.005
	Quadratic	1,17	1.065	0.317	0.059

Similar statistical results were obtained when the analysis was focused on the latency range of the Nd wave (98–128 ms) instead (cf. Table S3). The only difference was that there was a borderline significant quadratic effect in the ISI × attention interaction.

To directly compare the ISI dependence of the ARN and the (attended) N1, we normalized the amplitudes between experiments 1 and 2 and compared these conditions in an ANOVA for repeated measures. The analysis confirmed the strong ISI effect across the two experiments (*F*_1,17_ = 20.144; *p* < 0.001; $\eta_{p}^{2}$ = 0.542), while the interaction of experiment × ISI missed significance (*F*_2,34_ = 2.622; *p* = 0.087; $\eta_{p}^{2}$ = 0.134; general ANOVA; cf. Table S4 for full statistical analysis).

### 3.3 Longer Latency Range

The results of experiments 1 and 2 support the hypothesis that ARN and N1 amplitudes depend on the ISI, whereas the Nd—for the parameters used here—does not. The analysis reported so far was based on the first negative‐going peak in the grand average of both experiments. With respect to the dissociation of early and late components of the Nd in previous studies (Hansen and Hillyard 1984), we also evaluated a later time window. This is also of interest since the ARN showed different response behavior in an earlier and later time window in a previous study (Königs and Gutschalk 2012), and because the ARN appeared to be double peaked in some conditions of the grand average waveforms in the present study. In experiment 2, a second negative‐going peak is not so prominent compared to the N1, but a negative‐going sustained response is present here as well (cf. Figure 5A). To compare the second negative‐going response across conditions, the peak latency was determined in the grand average waveforms.

In experiment 1, the second negativity was then measured in the time interval of 180 to 210 ms (Figure S1) and showed overall similar effects of detection as observed in the early ARN time window (Table S1). Moreover, there were significant linear and quadratic interactions of ISI × detection, caused mostly by the detected condition.

In experiment 2, the measurement was performed in the time interval of 200–230 ms. Even though the broad‐based peak appeared more subtle here, it was of similar amplitude (around 10 nAm) as the second ARN peak measured in experiment 1 for the 500‐ms ISI. In contrast to the ARN in experiment 1, the amplitude decreased with increasing ISI showing significant linear and quadratic ISI effects in this condition (Table S2a). There was no significant difference between attended and unattended conditions in this time interval (Table S2a) ruling this part of the evoked response as not related to the Nd wave.

## 4 Discussion

These data confirm the hypothesis that the ARN evoked by detected, isochronous tone streams, shows ISI dependence. The objective to probe this response characteristic arose from the well‐known ISI dependence of the N1 response (Ritter et al. 1968; Hari et al. 1982; Imada et al. 1997). We further propose that the similarity of the two responses could mean that their ISI dependence relates to the perceptual organization of coherent auditory streams. In auditory stream segregation, a frequency‐specific suppression of the P1 and N1 response has been shown to be correlated with the tendency of two‐tone sequences to group into one or two streams (Gutschalk et al. 2005; Snyder et al. 2006). Previous psychoacoustic studies of multi‐tone masking have proposed a parallel to stream segregation before (Kidd Jr. et al. 1994, 2003; Micheyl et al. 2007) and a similar dependence on the frequency difference (widths of the protected region) has been reported for multi‐tone masking (Akram et al. 2014). If the ARN was closely related to the circuitry of the N1, it is conceivable that the amplitude of the response reflects the temporal relationship to other tones in the same stream, which would either be the previous tone in the target stream when detected, or the directly preceding tone of the multi‐tone masker when missed. Matching this interpretation, no amplitude increase with ISI was observed in the present study when the tone sequence remained masked.

In experiment 2, the N1 increment with increasing ISI was confirmed for similar stimulus sequences presented with a high‐frequency noise sequence but without a multi‐tone masker. This ISI effect was similar for attended and unattended versions of the same stimuli. Again, this finding would match an interpretation in terms of the perceptual organization, as the tone stream was never masked by or grouped with the narrow‐band noise stream in experiment 2, and thus it can be expected that its timing perception remained identical for attended and unattended presentations.

While these results show an ISI dependence for both, N1 and ARN, an amplitude increase beyond ISIs of 850 ms was only confirmed for the N1 in post hoc tests. Why the ARN could show less strong growth with the second ISI step remains unclear. Given the random character of the masker, the likelihood of a masker tone intruding into the target stream may be higher with higher ISI and could then also reduce the ARN amplitude. This would be plausible as the masker‐tone rate was constant across conditions. Since a target stream may well comprise other tones close by in frequency (Gärtner and Gutschalk 2021), the likelihood of such intrusions would be expected to increase with the ISI. Measuring such effects would require specific behavioral measures however, which are not available for the present data.

While previous studies had found variable modulation of the Nd with the ISI (Näätänen et al. 1981; Hansen and Hillyard 1984), the parameters used in the present study did not produce any systematic modulation of the Nd amplitude depending on the ISI. This finding further supports our hypothesis that the ARN is not simply identical with the Nd wave. Moreover, the Nd pattern found here was simpler than reported in previous studies (Hansen and Hillyard 1984; Rif et al. 1991). In particular, we could not dissociate early and late components of the Nd, but only a single component that overlapped the N1 evoked by unattended tones, already. As such, there was no clear dissociation of N1 and Nd unless difference waveforms were calculated. While activity in the latency range after the N1 was observed, this activity did not classify as Nd, as no difference between attended and unattended trials was observed. In contrast, the second peak of the ARN in experiment 1 showed overall similar behavior as the earlier one (i.e., stronger activity for hit than miss trials). The functional assignment of this longer‐latency component remains unclear. One possibility is that it could be a short sustained field evoked by the 100‐ms‐long tones; the auditory sustained fields (and potentials) have also been shown to increase with the ISI (Picton et al. 1978; Gutschalk, Patterson, et al. 2007), although a little less than the N1. One reason why this response was less prominent and did not increase with the ISI in experiment 2 could be because of cancellation with a more prominent P2 response evoked by unmasked sounds (cf. figure 4 in (Gutschalk et al. 2008)). Since P2 also increases with ISI (Hari et al. 1982), this could further explain the trend for smaller sustained negativity in experiment 2 with longer ISI (and maybe for undetected tones in experiment 1).

The peak latency of the early ARN around 120 ms was later than both the N1 and Nd in experiment 2, but the N1 latency has been previously reported to be stimulus and context sensitive; for example, the response latency increases when the sound intensity is reduced (Picton et al. 1976) or when the tones are presented in the presence of a noise masker (Billings et al. 2009). Therefore, the longer latency of the ARN compared to the N1 could simply be related to the presence of the multi‐tone masker and does not readily dissociate N1 and ARN.

The N1 evoked in quiet and without intramodal competition has traditionally been considered not to require attention (Näätänen and Picton 1987). More recently it has been demonstrated that the N1 is reduced when the load of a visual distractor is enhanced (Molloy et al. 2015), suggesting that some attentional drive of the N1 (or Nd) is present even in situations where the auditory stimuli are not task relevant. However, even in the high‐load condition, the majority of auditory stimuli were reported perceived when participants were asked for their presence in a dual‐task setup (Molloy et al. 2015). It is therefore unlikely, in our view, that the N1 as well as perceptual awareness are completely dependent on a top‐down attentional resource for salient stimuli and in situations without intramodal competition, while a major dependence on top‐down resources under intramodal, multi‐tone masking appears much stronger (Gutschalk et al. 2008). It is also the case that the detection of single, near‐threshold tones (under noise masking) requires task‐dependent attention and that a negative‐going evoked response is not observed for such stimuli when they are not task relevant (Doll et al. 2024). We therefore suggest that the N1/ARN relationship would better be in line with an intramodal framework like the biased‐competition model (Desimone and Duncan 1995) when considering that the N1 is relatively automatically evoked in a situation without intramodal competition but the ARN requires active attention in the presence of the multi‐tone masker. As such, the presence or lapse of a priori attention likely plays a role to determine whether a particular part of the scene is perceived or not (Sadaghiani et al. 2009; Doll et al. 2025), but we expect that it is not the pure presence of attention, but rather its direction towards the details required to bias the neural representation towards a certain perceptual organization in complex scenes like the ones used in the present study.

A potential functional relationship between the N1 and ARN would have implications beyond multi‐tone masking, because it could mean that the N1 may also reflect perceptual organization in simpler auditory scenes, where it is difficult to avoid an auditory percept (Wiegand et al. 2018). Meanwhile, parallels may extend to other negative‐going long‐latency components generated in the auditory cortex: a similar negative‐going response evoked by solitary detected tones has been referred to as auditory awareness negativity (AAN) (Dembski et al. 2021; Schlossmacher et al. 2021). The labels ARN and AAN have lately been used interchangeably (Dembski et al. 2021); note however that ARN was not conceptualized as a difference response (Gutschalk et al. 2008), whereas AAN has been reported as a difference response between detected and undetected tones (Eklund and Wiens 2019). The object‐related negativity (ORN) is evoked when two concurrent sounds are segregated, for example in the case of a mistuned harmonic, and appears to be similarly related to the perception of this tone standing out from its background as the ARN and AAN (Alain et al. 2002; Gohari et al. 2022), with the difference that the common onset of the object and background makes it more difficult to assign the ORN unambiguously to the object. Finally, the MMN has been previously shown to operate on perceived streams rather than physically defined sound sequences (Sussman et al. 1999; Winkler et al. 2003). The MMN stands distinct in this list, however, as it is defined relative to the response evoked by a standard that has supposedly been perceived as well.

The neural underpinnings of these negative‐going responses in auditory cortex are only incompletely understood. Based on laminar recordings (Javitt et al. 1996; Lakatos et al. 2013), long‐latency, negative‐going evoked responses are related to synaptic drive in the upper layers of the auditory cortex. Current biophysical models of auditory‐evoked fields further suggest that the N1 and ARN (Kohl et al. 2022; Fernandez Pujol et al. 2023) are related to currents in layer 5 pyramidal neurons, with no major difference predicted between N1 and ARN. This finding would fit with recent findings that layer 5 pyramidal neurons that project to subcortical targets are required for detection of peri‐threshold somatosensory stimuli in a mouse model (Takahashi et al. 2020). What the biophysical models cannot capture, however, are the sources that drive the upper‐layer synaptic input. It is therefore possible that the N1, Nd, and ARN may be distinct in the sources of neural input and connectivity, even when they likely converge into a similar activation of layer 5 pyramidal neurons. One hypothetical scenario could be, for example, that the N1 receives predominantly input from belt and para‐belt auditory cortex, whereas Nd could receive more input from supra‐modal areas controlling top‐down attention. In that case, we would predict that the ARN recorded under informational masking receives coherent input from both sources.

## Author Contributions

**Clara Raudonat:** conceptualization, investigation, formal analysis, data curation, writing – original draft. **Eva Doroszewski:** conceptualization, software, formal analysis, writing – review and editing. **Alexander Gutschalk:** conceptualization, supervision, validation, writing – review and editing, funding acquisition.

## Funding

Deutsche Forschungsgemeinschaft grant DFG 593/5‐1 (AG).

## Conflicts of Interest

The authors declare no conflicts of interest.

## Supporting information


**Appendix S1:** psyp70198‐sup‐0001‐AppendixS1.docx.

## Data Availability

The MEG data are available on heiDATA, the open research data repository of Heidelberg University: https://doi.org/10.11588/DATA/UWW2LJ.
