# Supplementary material for: Inter‐Stimulus‐Interval Dependence in Auditory Cortex Reflects Perceptual Organization of Streams Under Informational Masking
Source: Psychophysiology. 2025 Nov 30;62(12):e70198. doi: 10.1111/psyp.70198 (PMC12665807; doi:10.1111/psyp.70198)
Supplement: Supplementary file 1 — Appendix S1: psyp70198‐sup‐0001‐AppendixS1.docx. [file PSYP-62-e70198-s001.pdf]

Supplementary Figures and Tables

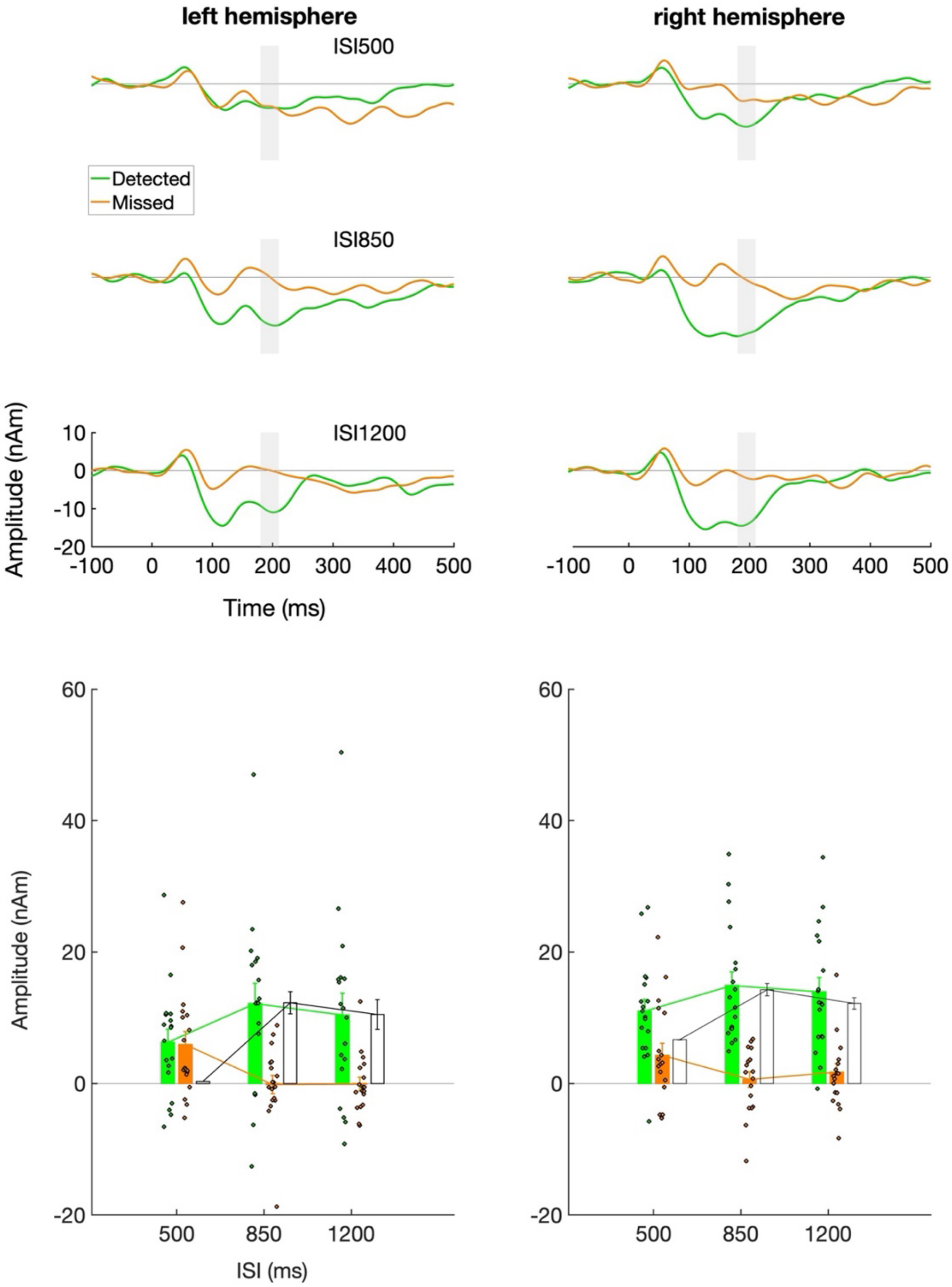

Figure S1. **(A)** Grand average (n=18) source waveforms of Experiment 1 for dipoles in left and right auditory cortex. The three ISI conditions (500 ms, 850 ms, and 1200 ms) are plotted on the x axis. The source waveforms are separately plotted for detected (green) and undetected (orange) trials. The time interval in which the ARN amplitude was measured for the analysis (180-210 ms) is shaded in gray. **(B)** Mean (± standard error) amplitudes in the left and right auditory-cortex source waveforms across participants (n=18) in the ARN interval (180-210 ms) for experiment 1; single participant amplitudes indicated as small circle. The values are separately plotted for hit (green) and miss (orange) trials over the three ISI conditions used.

Table S1. Statistical analysis of Experiment 1. (a) ANOVA for repeated measures for the ARN amplitude in the source waveforms in a later time window (180-210 ms) with the factors detection (detected, undetected), ISI (500, 850, 1200 ms; linear contrast analysis), and hemisphere (left, right) (b) ANOVA for detected tones, only (c) ANOVA for undetected tones, only.

(a) Detected vs. undetected	*Contrast*	***df***	***F***	***Sig.***	η^2_p
Detection		1,17	32.453	<0.001	0.656
ISI	Linear	1,17	0.205	0.656	0.012
	Quadratic	1,17	0.031	0.863	0.002
Hemisphere		1,17	3.498	0.079	0.171
Det*ISI	Linear	1,17	9.689	0.006	0.363
	Quadratic	1,17	13.356	0.002	0.440
Det*Hemisphere		1,17	3.875	0.066	0.186
Det*ISI*Hemisphere	Linear	1,17	2.500	0.132	0.128
	Quadratic	1,17	0.876	0.362	0.049
(b) Detected					
ISI	Linear	1,17	7.904	0.012	0.317
	Quadratic	1,17	11.503	0.003	0.404
Hemisphere		1,17	4.599	0.047	0.213
ISI*Hemisphere	Linear	1,17	0.503	0.488	0.029
	Quadratic	1,17	0.723	0.407	0.041
**(c) *Undetected***					
ISI	Linear	1,17	6.372	0.002	0.273
	Quadratic	1,17	2.954	0.104	0.148
Hemisphere		1,17	0.127	0.725	0.007
ISI*Hemisphere	Linear	1,17	1.779	0.200	0.095
	Quadratic	1,17	0.264	0.614	0.016

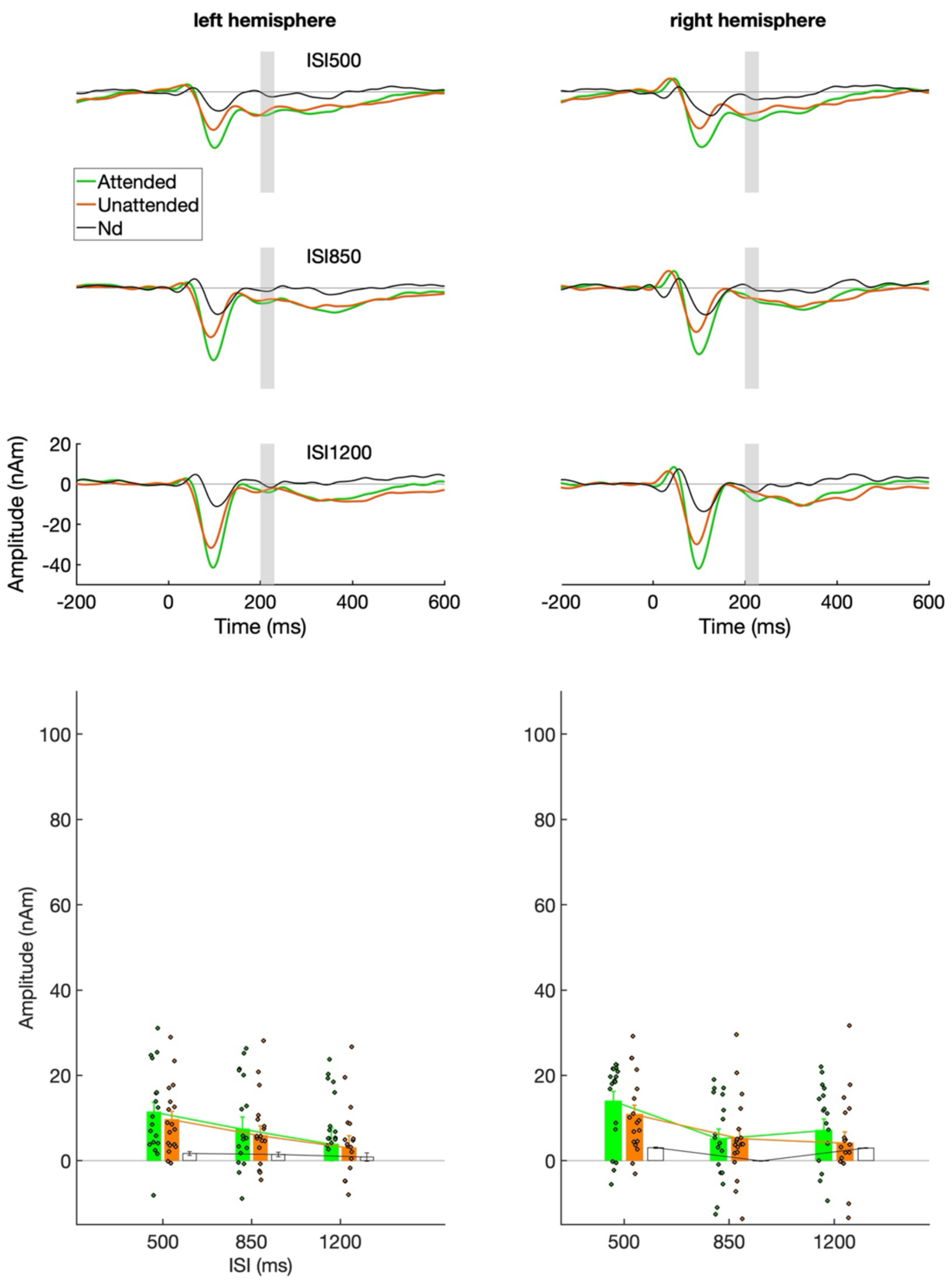

Figure S2. (A) Grand average (n=18) source waveforms of Experiment 2 for dipoles in left and right auditory cortex. The three ISI conditions (500 ms, 850 ms, and 1200 ms) are plotted in a column. The source waveforms are separately plotted for attended (green) and unattended (orange) trials. The difference of attended minus unattended is blotted in black. The time interval in which the N1 amplitude was measured for the analysis (200-230 ms) is shaded in gray. **(B)** Mean (± standard error) N1 amplitudes in the left and right auditory-cortex source waveforms across participants (n=18) in experiment 2 (200-230 ms); single participant amplitudes indicated as small circle. The values are separately plotted for attended (green) and unattended (orange) trials over the three ISI conditions used. Mean difference amplitudes are indicated as white bars.

Table S2. Statistical analysis of Experiment 2. (a) ANOVA for repeated measures for the amplitude in the source waveforms for the late negative-going wave (200-230 ms) following N1 with the factors attention (attended, unattended), ISI (500, 850, 1200 ms; linear contrast analysis), and hemisphere (left, right) (b) ANOVA for attended tones, only (c) ANOVA for uunattended tones, only.

(a) Attended vs. unattended	*Contrast*	*df*	*F*	*Sig.*	η^2_p
Attention		1,17	0.612	0.445	0.035
ISI	Linear	1,17	16.578	<0.001	0.494
	Quadratic	1,17	9.571	0.007	0.360
Hemisphere		1,17	0.399	0.536	0.023
Att*ISI	Linear	1,17	0.088	0.771	0.005
	Quadratic	1,17	1.720	0.207	0.092
Att*Hemisphere		1,17	0.231	0.637	0.013
Att*ISI*Hemisphere	Linear	1,17	0.135	0.718	0.008
	Quadratic	1,17	8.333	0.010	0.329
(b) Attended					
ISI	Linear	1,17	12.377	0.003	0.421
	Quadratic	1,17	6.787	0.018	0.285
Hemisphere		1,17	0.612	0.445	0.035
ISI*Hemisphere	Linear	1,17	0.065	0.801	0.004
	Quadratic	1,17	22.893	<0.001	0.574
**(c) *Unattended***					
ISI	Linear	1,17	15.741	<0.001	0.481
	Quadratic	1,17	5.289	0.034	0.237
Hemisphere		1,17	0.128	0.725	0.007
ISI*Hemisphere	Linear	1,17	0.001	0.971	0.000
	Quadratic	1,17	5.258	0.035	0.236

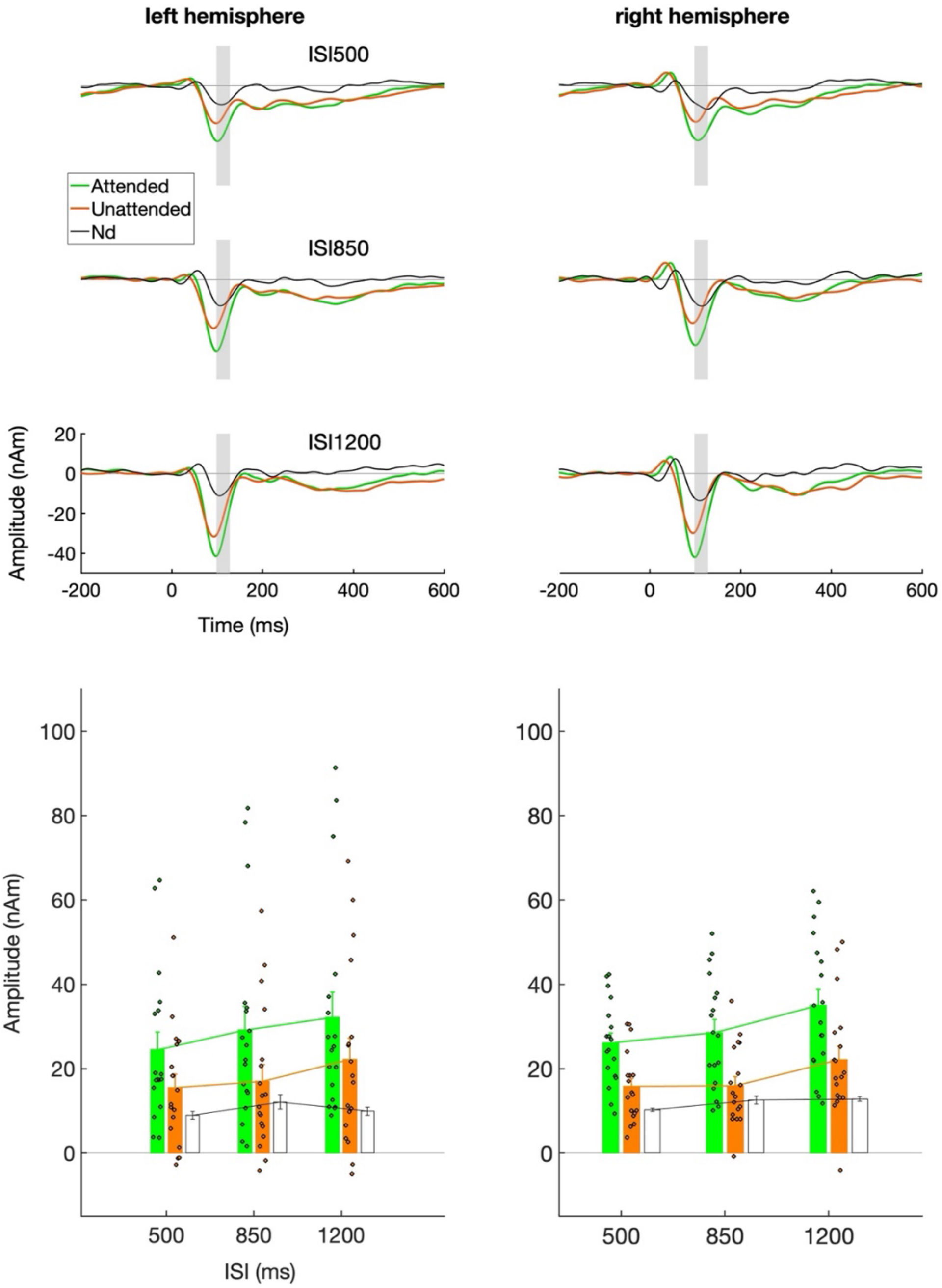

Figure S3. (A) Grand average (n=18) source waveforms of Experiment 2 for dipoles in left and right auditory cortex. The three ISI conditions (500 ms, 850 ms, and 1200 ms) are plotted in a column. The source waveforms are separately plotted for attended (green) and unattended (orange) trials. The difference of attended minus unattended is blotted in black. The time interval in which the N1 amplitude was measured for the analysis (98-128 ms) is shaded in gray. **(B)** Mean (± standard error) N1 amplitudes in the left and right auditory-cortex source waveforms across participants (n=18) in experiment 2 (98-128 ms); single participant amplitudes indicated as small circle. The values are separately plotted for attended (green) and unattended (orange) trials over the three ISI conditions used. Mean difference amplitudes are indicated as white bars.

Table S3. Statistical analysis of Experiment 2. (a) ANOVA for repeated measures for in the source waveforms in a specific Nd time window (98-128 ms) with the factors attention (attended, unattended), ISI (500, 850, 1200 ms; linear contrast analysis), and hemisphere (left, right) (b) ANOVA for attended tones, only (c) ANOVA for uunattended tones, only.

(a) Attended vs. Unattended (98-128 ms)	*Contrast*	*df*	*F*	*Sig.*	η^2_p
Attention		1,17	24.030	<0.001	0.586
ISI	Linear	1,17	12.074	0.003	0.415
	Quadratic	1,17	4.454	0.050	0.208
Hemisphere		1,17	0.023	0.880	0.001
Attention*ISI	Linear	1,17	1.109	0.307	0.061
	Quadratic	1,17	3.276	0.088	0.162
Attention*Hemisphere		1,17	0.979	0.336	0.054
Attention*ISI*Hemisphere	Linear	1,17	0.707	0.412	0.040
	Quadratic	1,17	1.129	0.303	0.062
(b) Attended					
ISI	Linear	1,17	12.613	0.002	0.426
	Quadratic	1,17	0.308	0.586	0.018
Hemisphere		1,17	0.142	0.711	0.008
**ISI*Hemisphere**	Linear	1,17	0.361	0.556	0.021
	Quadratic	1,17	6.369	0.022	0.273
(c) Unattended					
ISI	Linear	1,17	8.413	0.010	0.331
	Quadratic	1,17	13.376	0.002	0.440
Hemisphere		1,17	0.006	0.937	0.000
ISI*Hemisphere	Linear	1,17	0.065	0.802	0.004
	Quadratic	1,17	1.383	0.256	0.075

Table S4. Statistics comparison of the ISI effect between Experiments 1 and 2. Amplitudes of ARN and N1 (attended) were normalized for each participant such that the average amplitude within each ARN and each N1 data set was 1. (a) ANOVA for repeated measures with the factors Experiment (1: ARN, 2: N1-attended), ISI (500, 850, and 1200 ms), and hemisphere (left, right) (b) ANOVA limited to the first ISI interval (500 versus 850). (c) ANOVA limited to the second ISI interval (850 versus 1200). Because of the normalization, there is absolutely no Experiment effect. The interaction of ARN/N1 * ISI shows a non-significant statistical trend, which is caused by a just significant difference in the first ISI interval, but note that the post-hoc test in b and c are not corrected for multiple comparison.

(a) ARN vs. N1-attended	*df*	*F*	*Sig.*	η^2_p
**Experiment (ARN/N1)**	1,17	0.000	1.000	0.000
ISI	2,34	20.144	<0.001	0.542
Hemisphere	1,17	0.270	0.610	0.016
**ARN/N1***ISI	2,34	2.622	0.087	0.134
**ARN/N1***Hemisphere	1,17	2.618	0.124	0.134
**ARN/N1***ISI*Hemisphere	2,34	1.821	0.177	0.100
**(b) ISI 500 vs. ISI850**				
ISI	1,17	15.078	0.001	0.470
Hemisphere	1,17	0.486	0.495	0.028
**ARN/N1***ISI	1,17	4.491	0.049	0.209
**ARN/N1***Hemisphere	1,17	4.278	0.054	0.201
**ARN/N1***ISI*Hemisphere	1,17	1.005	0.330	0.055
**(c) ISI 850 vs. ISI1200**				
ISI	1,17	7.334	0.015	0.302
Hemisphere	1,17	0.165	0.689	0.010
**ARN/N1***ISI	1,17	0.833	0.374	0.047
**ARN/N1***Hemisphere	1,17	2.114	0.164	0.110
**ARN/N1***ISI*Hemisphere	1,17	3.689	0.072	0.178
